# A Rare Case of Spindle Cell Neoplasm of the Left Leg Managed With Wide Local Excision and Split-Thickness Skin Grafting

**DOI:** 10.7759/cureus.97401

**Published:** 2025-11-21

**Authors:** Bhavya Sinha, Barathi Raja Kuppusami, N Guru Prasad, Samir Ahmad, Santhaseelan R G

**Affiliations:** 1 General Surgery, Sree Balaji Medical College and Hospital, Chennai, IND

**Keywords:** dermatofibrosarcoma protuberans, extremity sarcoma, skin grafting, soft tissue tumor, spindle cell neoplasm, wide local excision

## Abstract

Spindle cell neoplasms are a diverse category of tumors of the soft tissue that have disparate histological complexity and malignancy. Dermatofibrosarcoma protuberans, a dermal spindle cell tumor, is a rare but locally destructive neoplasm with high potential for recurrence once incompletely removed. We report the case of a 25-year-old female with a spindle cell neoplasm in the left leg, successfully treated with wide local excision and split-thickness skin grafting. The nature of the tumor was spindle cell, confirmed by histopathology, and the surgical margins were clear. This case emphasizes the significance of proper margin control in spindle cell sarcoma, considers reconstructive factors, and describes the present literature related to the diagnosis and management.

## Introduction

Fewer than 1% of adult malignancies are soft tissue sarcomas, and spindle cell variations lead to diagnostic and treatment challenges because of their diverse histology and characteristics that overlap with benign mimics [1,2]. Dermatofibrosarcoma protuberans (DFSP), one such spindle cell tumor, is classified as a low-to-intermediate-grade sarcoma with high local recurrence but low metastatic potential [3,4]. It commonly manifests in young people as a hard, slowly growing plaque or nodular mass on the trunk and extremities. Histologically, DFSP has CD34 immunoreactivity and is distinguished by homogeneous spindle cells grouped in a storiform pattern [5]. Molecularly, more than 90% harbor a t(17;22)(q22;q13) translocation resulting in *COL1A1-PDGFB* fusion, which drives PDGFβ receptor activation and provides a rationale for targeted therapy [5]. Surgery remains the gold standard of treatment [6]. To reduce recurrence, Mohs micrographic surgery or wide local excision (WLE) with 2-3 cm tumor-free margins is advised [1,6]. Depending on the size and location of the lesion, skin grafting or flap covering is frequently necessary for reconstruction after radical excision. We report the case of a young female with a spindle cell neoplasm of the leg managed successfully with WLE and split skin grafting, reviewing its diagnostic challenges and therapeutic principles.

## Case presentation

**Figure 1:**
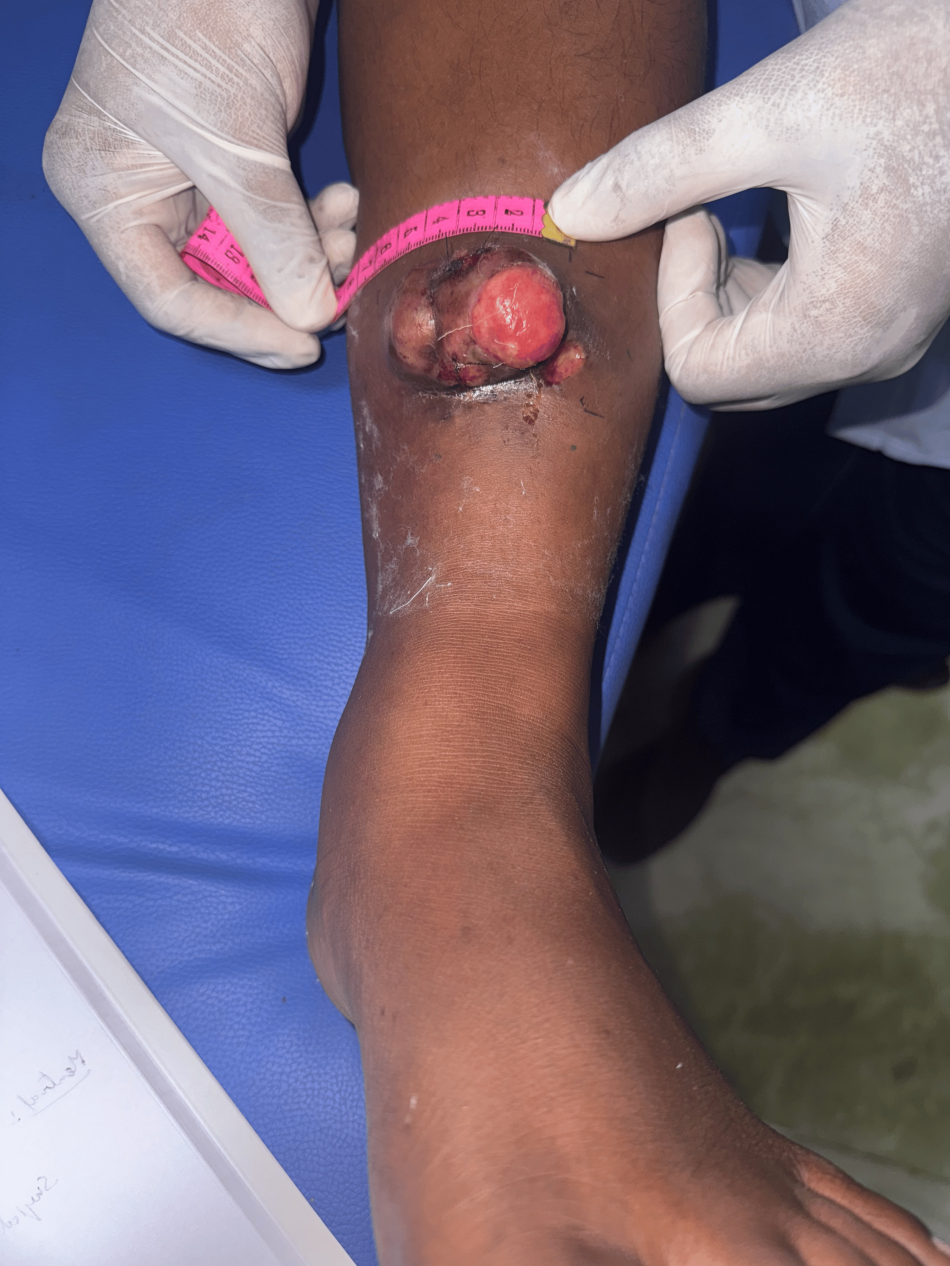
Preoperative image showing a lobulated exophytic mass over the anteromedial aspect of the left leg, measuring approximately 5 × 5 cm, with excoriated overlying skin.

**Figure 2:**
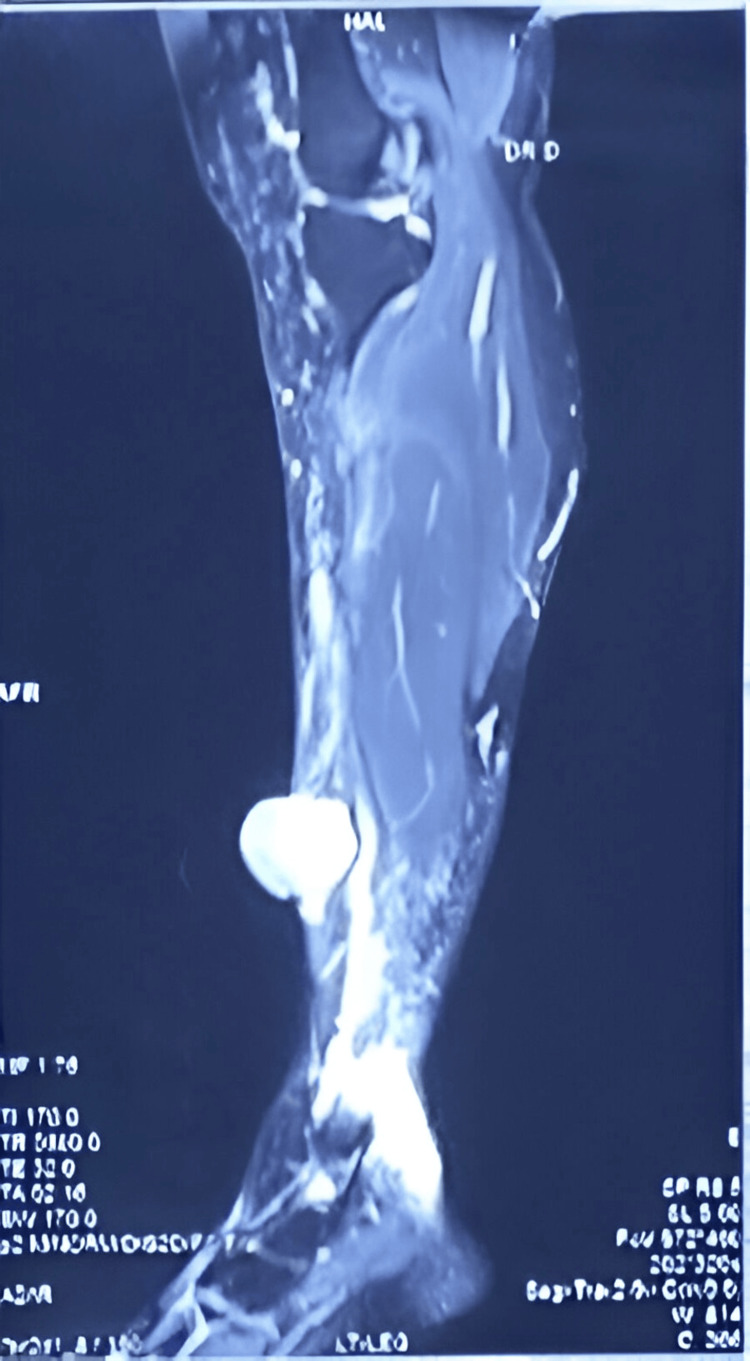
Preoperative MRI of the left leg showing a lesion measuring 3.4 × 9.2 × 3.2 cm in the subcutaneous plane, sparing the tibia, fibula, muscle, and neurovascular bundle.

A 25-year-old woman, G3P1L1A1, presented with an eight-year history of progressive swelling over the left leg. She had a prior history of excision at the same site 10 years ago. The swelling recurred at the same site. There was no history of weight loss, fever, or systemic illness. She was currently breastfeeding and postpartum. The patient has no comorbidities or family history of any malignancy. On examination, a solitary 5 × 5 cm lobulated swelling was noted over the anteromedial aspect of the left leg, 10 cm above the ankle. The skin over the swelling was excoriated, with no discharge or signs of infection (Figure 1). The swelling was mobile, non-tender, firm, with well-defined edges, and no inguinal lymphadenopathy. Pulses were palpable in all peripheral vessels. MRI of the left leg revealed a lesion measuring 3.4 × 9.2 × 3.2 cm in the subcutaneous plane, sparing the tibia, fibula, muscle, and neurovascular bundle (Figure 2).

**Figure 3:**
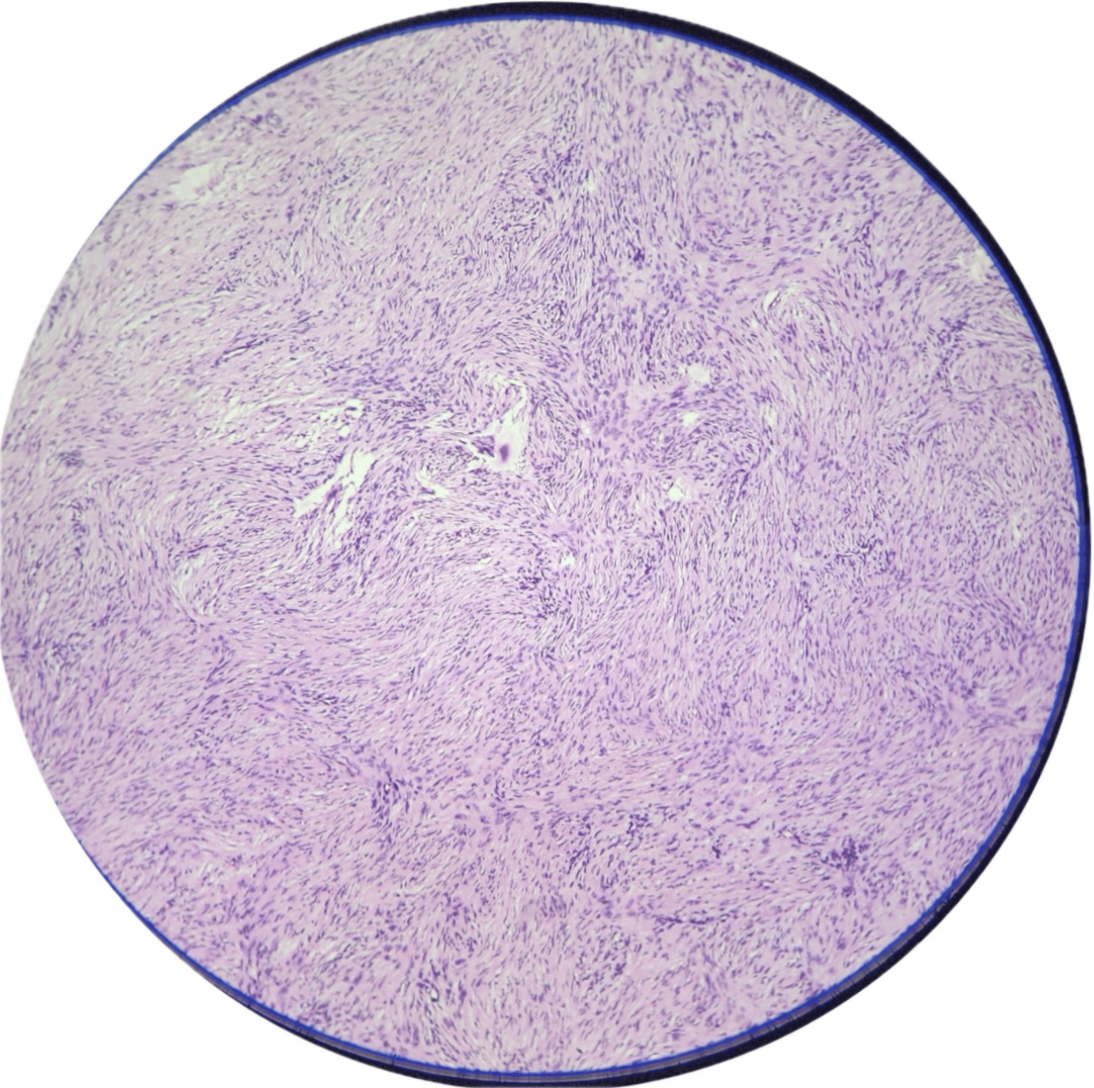
10× view on microscopy showing spindle cells arranged in a storiform pattern.

**Figure 4:**
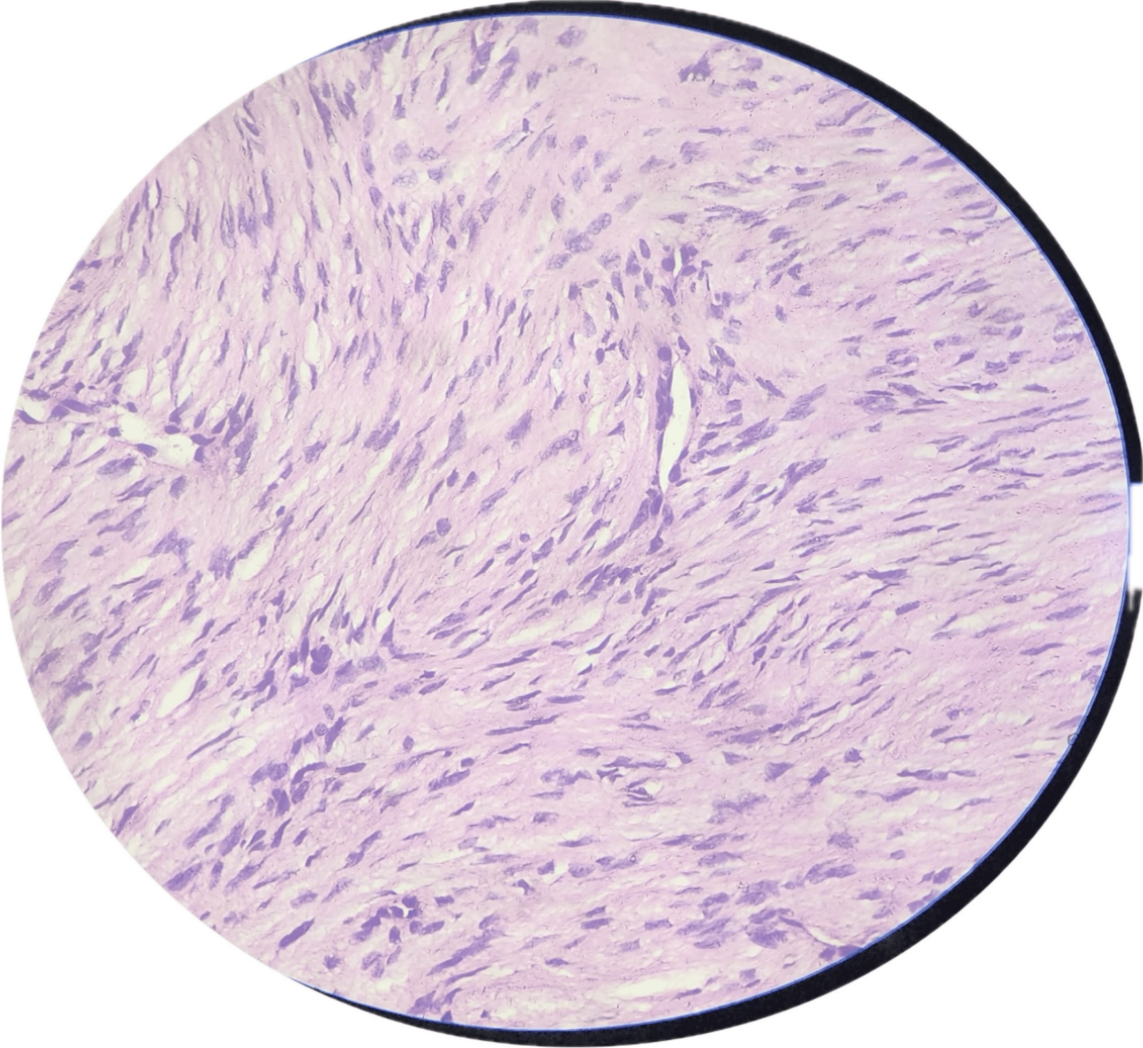
High-power (40×) view on microscopy showing spindle-shaped cells with slender nuclei and moderate amount of cytoplasm.

**Figure 5:**
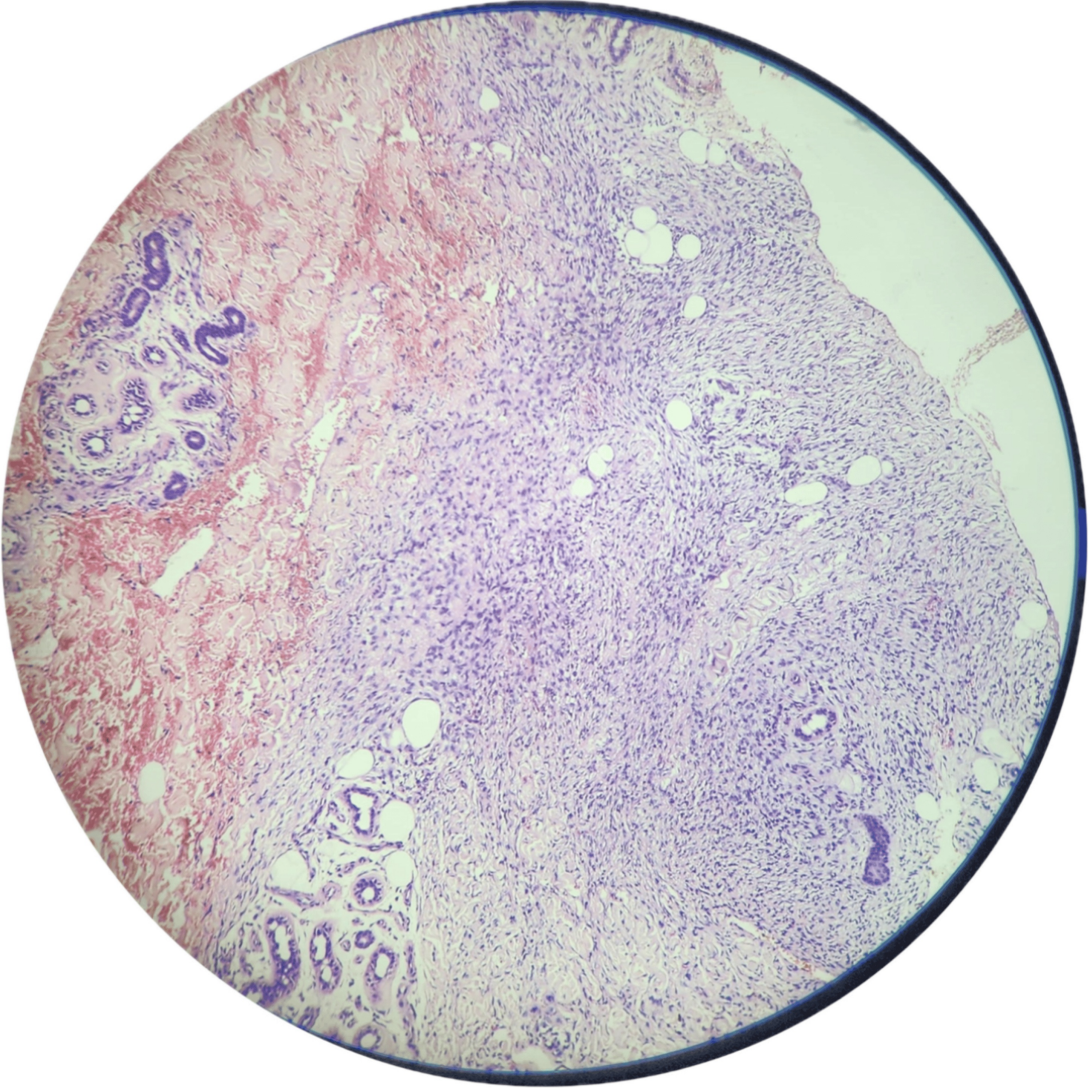
High-power view in microscopy showing the same spindle cell neoplasm in the subcutis.

**Figure 6:**
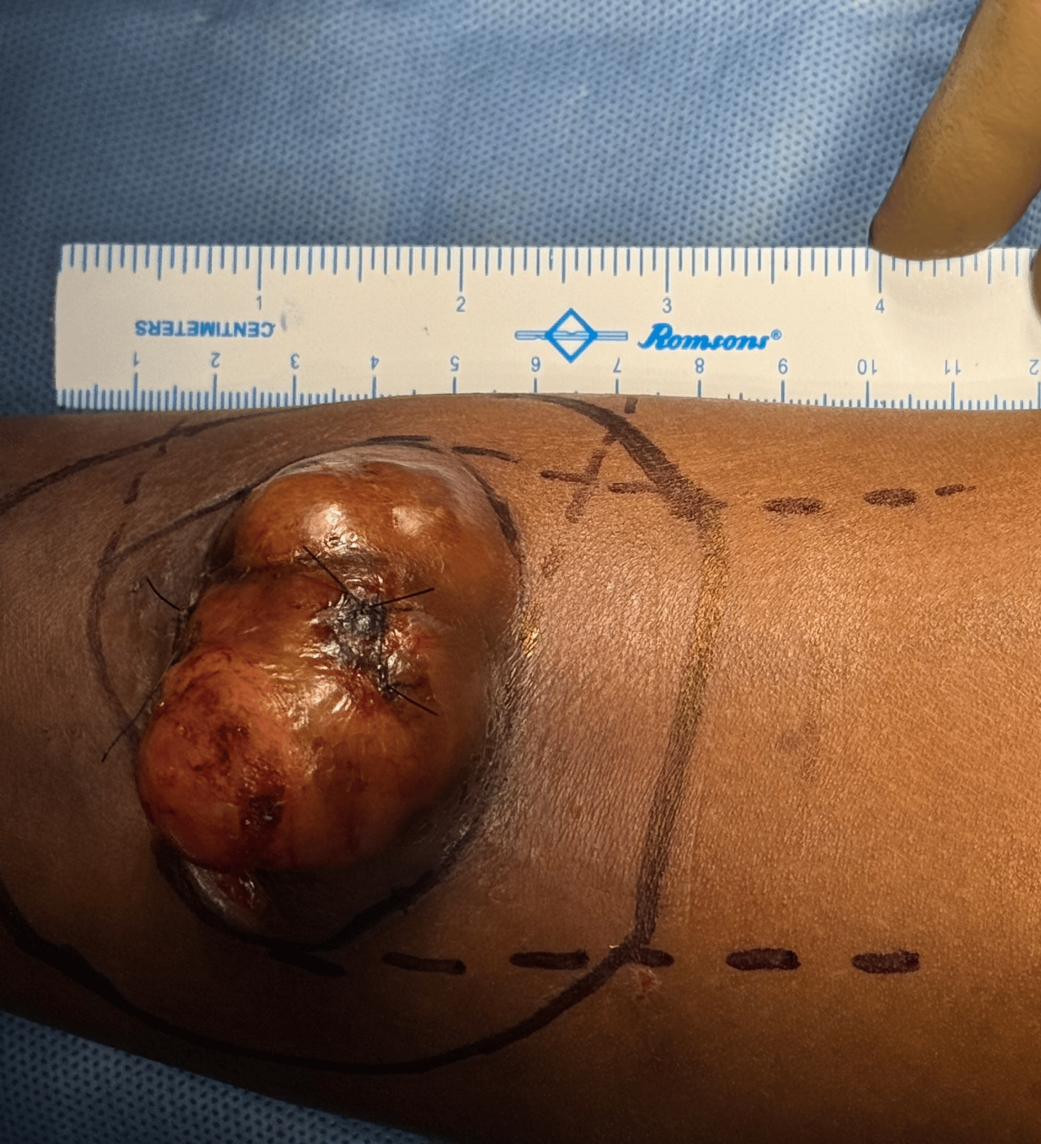
Operative field showing surgical markings for planned wide local excision with oncological margins.

**Figure 7:**
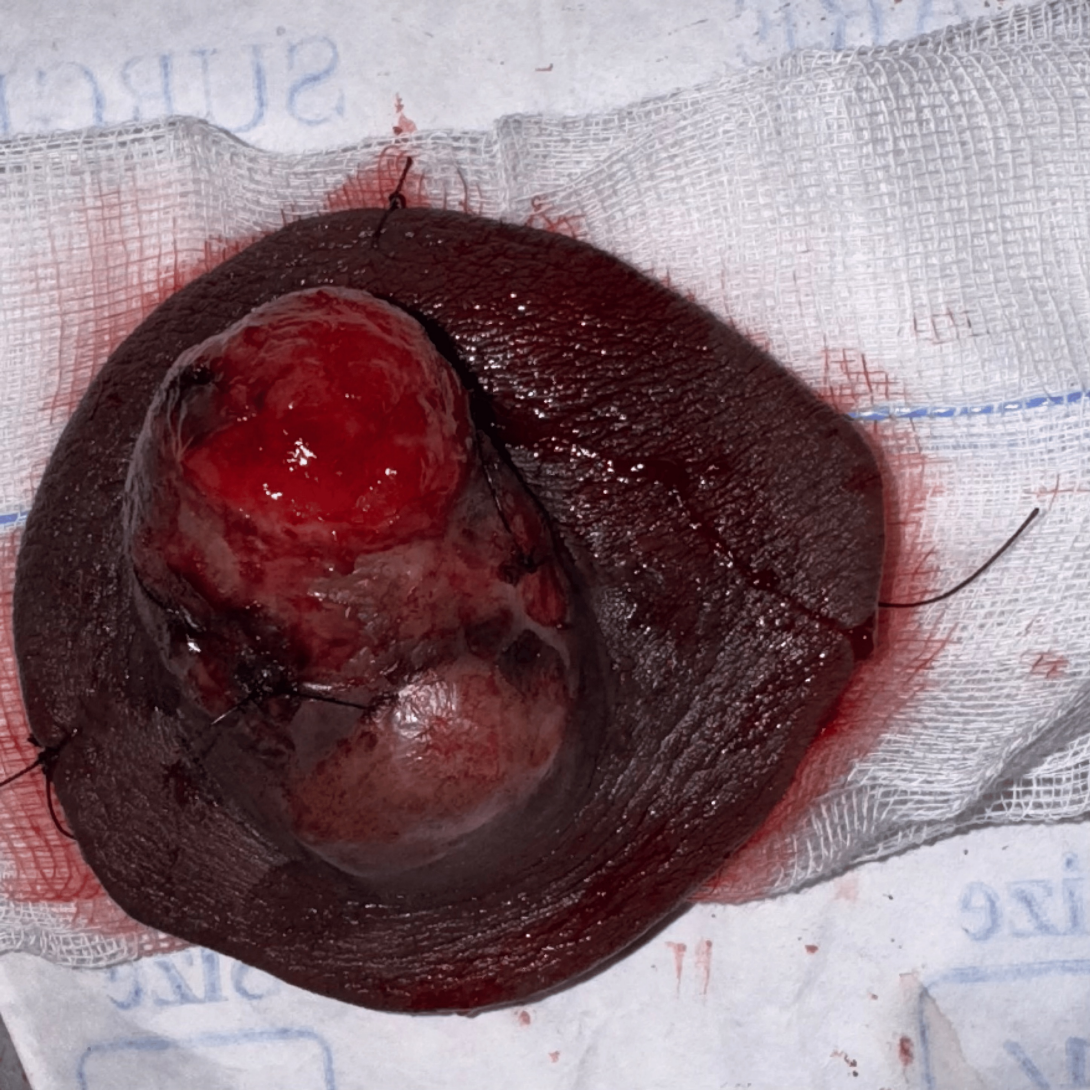
Gross specimen showing nodular, encapsulated soft tissue tumor with overlying skin ellipse.

**Figure 8:**
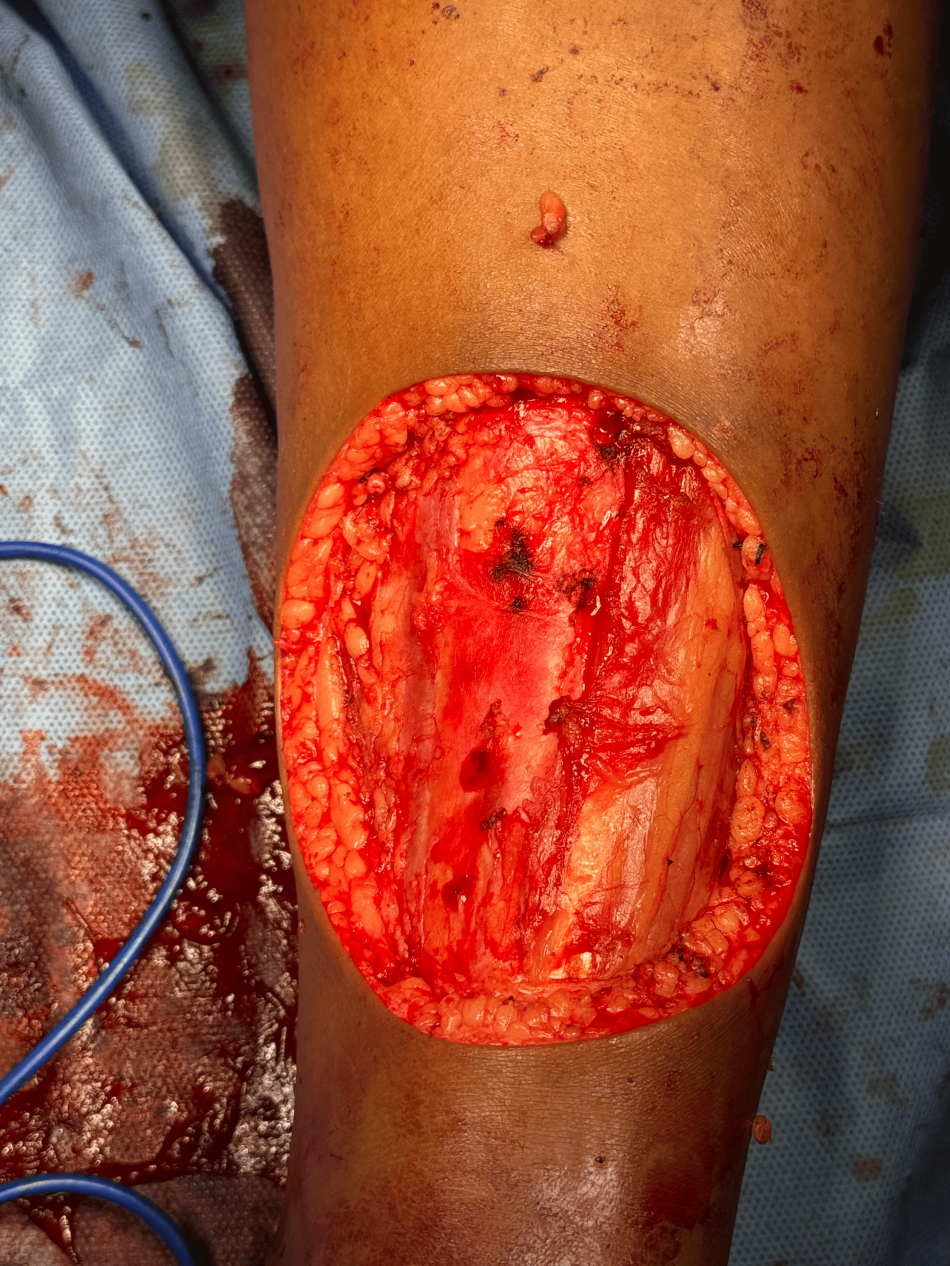
Tumor bed post-excision showing subcutaneous tissue and exposed fascia, with all margins cleared.

A biopsy resulted in a diagnosis of a spindle cell neoplasm. Histopathology suggested a tumor composed of slender spindle cells in whorls and sheets with no abnormal mitosis (Figures 3-5). Tumor-free margins confirmed a spindle cell neoplasm, likely DFSP. Immunohistochemistry (IHC) markers advised CD34, Ki-67, SOX10, and S100. WLE was done, which covered the underlying deep fascia: superior margin, 2 cm away from the tumor; lateral margin, 2 cm away from the tumor; medial margin, 3 cm away from the tumor; inferior margin, 2 cm away from the tumor (Figures 6-8).

**Figure 9:**
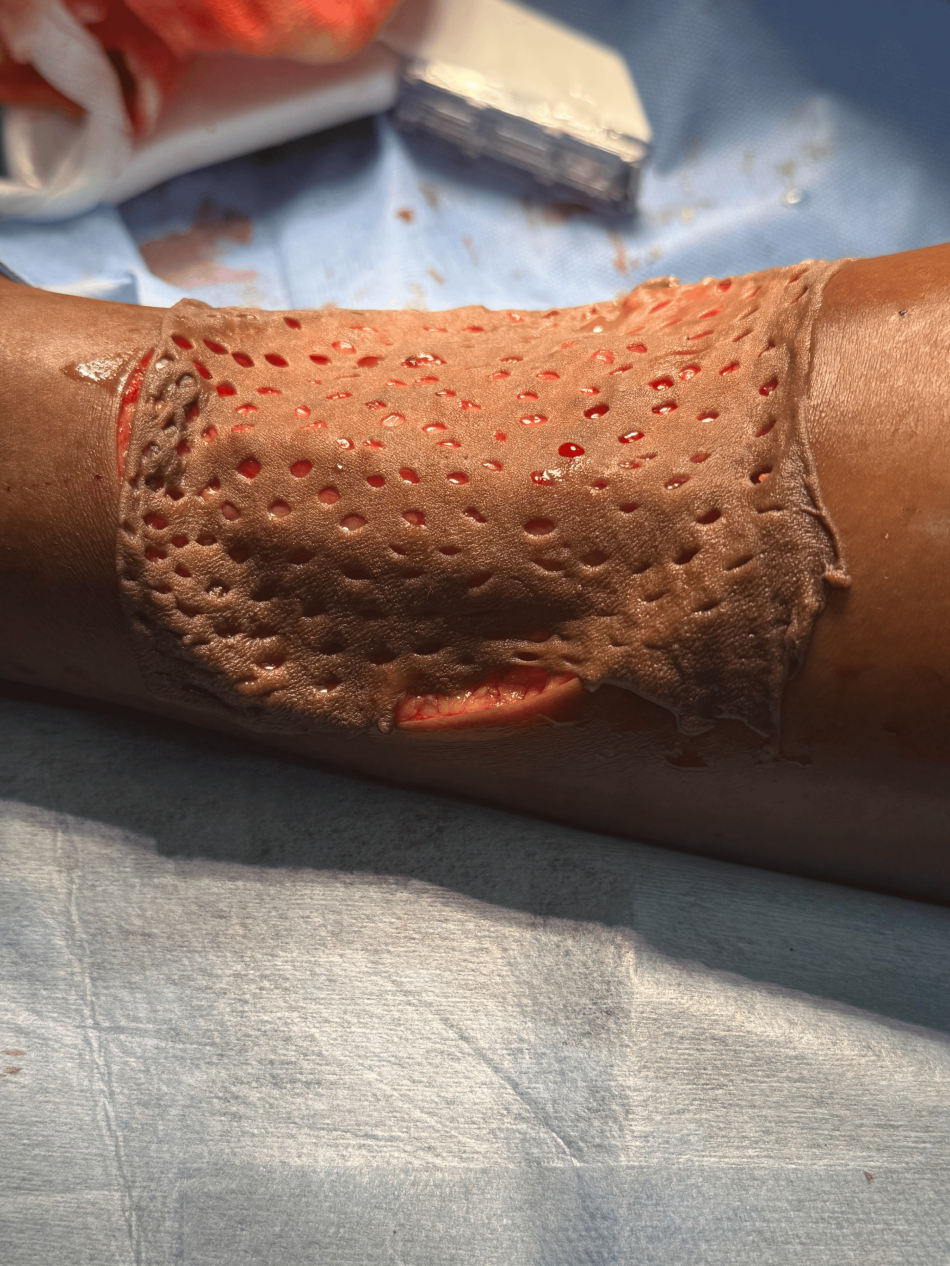
Split-thickness skin graft placed over the defect site and secured after hemostasis.

**Figure 10:**
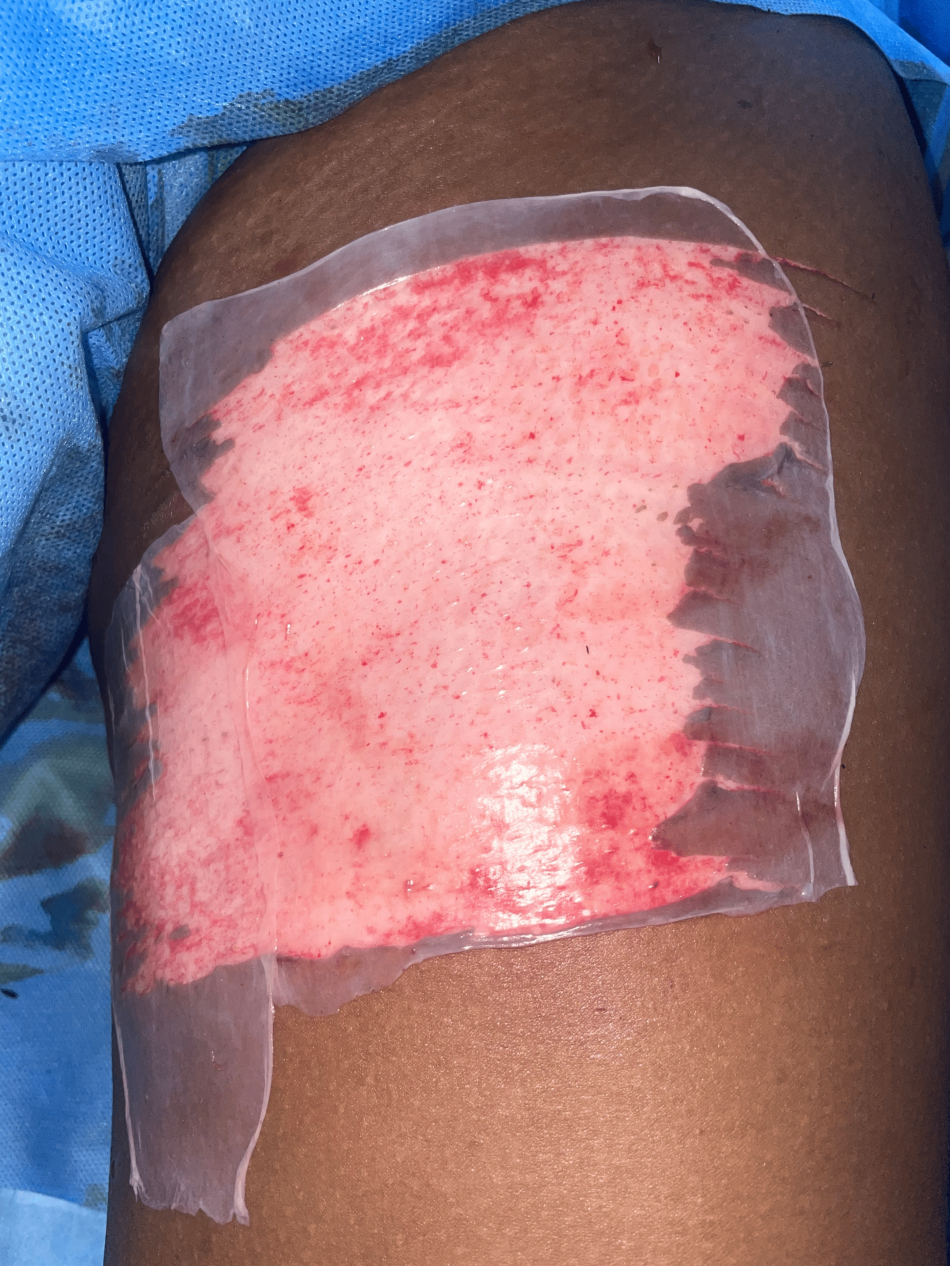
Collagen sheet applied to the graft donor site (anteromedial thigh) post-harvest for enhanced healing.

**Figure 11:**
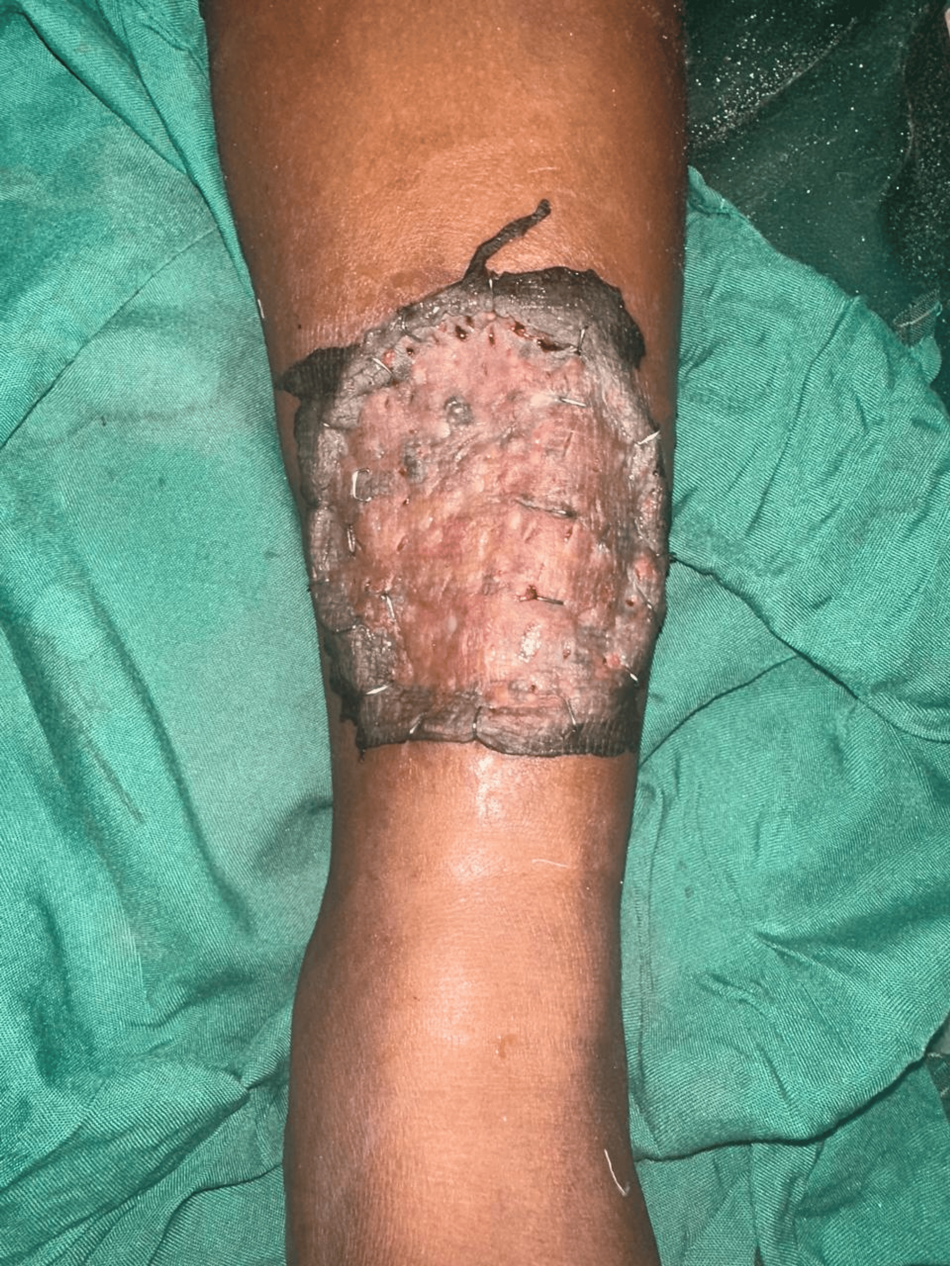
Postpperative image showing graft uptake of more than 90% after one week.

A split-thickness skin graft was harvested from the anteromedial aspect of the left thigh (Figure 9). In the intraoperative frozen section, margins were confirmed to be free of tumor. A collagen sheet was applied to the donor site (Figure 10). The graft was secured with staples, and the limb was immobilized in a below-knee plaster of Paris slab. Postoperative recovery was uneventful. No signs of graft rejection or infection were noted (Figure 11). The IHC report suggested Ki67 of 2-3% and CD34 diffuse cytoplasmic positivity. The patient was advised to have regular follow-up 12 months post-surgery with oncology and surgical teams. Follow-up revealed no evidence of recurrence. The patient was started on adjuvant external beam radiation therapy at around 55-60 Gy over five to six weeks.

## Discussion

Spindle cell tumors of the extremities encompass a broad differential diagnosis, including solitary fibrous tumor, DFSP, fibrosarcoma, synovial sarcoma, and malignant peripheral nerve sheath tumors [7]. IHC serves a pivotal function in narrowing the diagnosis. DFSP typically exhibits strong CD34 positivity, while other markers, such as S100, SOX10, and STAT6, help exclude neural or solitary fibrous tumor variants [5,7]. Adequate margin control is paramount in preventing local recurrence. Kawaguchi et al. proposed a classification where curative margins require ≥3-5 cm clearance, with wide margins of ≥2 cm considered acceptable in responsive tumors [1]. The Scandinavian Sarcoma Group studies also emphasize that positive or close margins correlate with recurrence, reinforcing the need for precise histopathologic assessment [6]. DFSP carries recurrence rates of up to 50-60% if margins are inadequate [4]. Compared to traditional WLE, Mohs micrographic surgery has demonstrated a decreased recurrence rate, especially in anatomically limited locations [5]. According to the National Comprehensive Cancer Network/European Society for Medical Oncology guidelines, WLE has a 10-60% recurrence rate, whereas Mohs micrographic surgery has a less than 2-5% recurrence rate.. However, in extremities, WLE with skin grafting remains a safe and effective option. While surgery suffices in most cases, radiotherapy may be considered for close margins or unresectable disease [1,6]. Targeted therapy with imatinib mesylate is reserved for unresectable, recurrent, or metastatic DFSP harboring *COL1A1-PDGFB* fusion [5]. The young female patient in our case underwent WLE with clear margins and immediate reconstruction using split-thickness skin grafting, restoring limb integrity and minimizing functional impairment. Early follow-up showed good graft uptake without recurrence. Similar reconstructive strategies have been reported to be effective in large or recurrent DFSP defects [8].

## Conclusions

The presentation of DFSP is usually delayed due to slow growth. This case presentation highlights the significance of early clinical suspicion, appropriate radiological evaluation, as well as complete surgical excision in the management of spindle cell neoplasms. In this case, 12 months of follow-up showed no evidence of local recurrence, and the graft site was healthy. MRI is essential for preoperative planning. DFSP, though rare, should be considered in longstanding soft tissue swellings. Multidisciplinary coordination and regular follow-up are essential to monitor recurrence.
